# Terrestrial laser scanning: a new standard of forest measuring and modelling?

**DOI:** 10.1093/aob/mcab111

**Published:** 2021-09-06

**Authors:** Markku Åkerblom, Pekka Kaitaniemi

**Affiliations:** 1 Unit of Computing Sciences, Tampere University, FI-33014 Tampere University, Finland; 2 Hyytiälä Forestry Field Station, Faculty of Agriculture and Forestry, University of Helsinki, Hyytiäläntie 124, FI-35500 Korkeakoski, Finland

**Keywords:** Forest mensuration, terrestrial laser scanning, data processing, comprehensive tree reconstruction, quantitative structure modelling, forest process research

## Abstract

**Background:**

Laser scanning technology has opened new horizons for the research of forest dynamics, because it provides a largely automated and non-destructive method to rapidly capture the structure of individual trees and entire forest stands at multiple spatial scales. The structural data themselves or in combination with additional remotely sensed data also provide information on the local physiological state of structures within trees. The capacity of new methods is facilitated by the ongoing development of automated processing tools that are designed to capture information from the point cloud data provided by the remote measurements.

**Scope:**

Terrestrial laser scanning (TLS), performed from the ground or from unmanned aerial vehicles, in particular, has potential to become a unifying measurement standard for forest research questions, because the equipment is flexible to use in the field and has the capacity to capture branch-level structural information at the forestplot or even forest scale. This issue of *Annals of Botany* includes selected papers that exemplify the current and potential uses of TLS, such as for examination of crown interactions between trees, growth dynamics of mixed stands, non-destructive characterization of urban trees, and enhancement of ecological and evolutionary models. The papers also present current challenges in the applicability of TLS methods and report recent developments in methods facilitating the use of TLS data for research purposes, including automatic processing chains and quantifying branch and above-ground biomass. In this article, we provide an overview of the current and anticipated future capacity of TLS and related methods in solving questions that utilize measurements and models of forests.

**Conclusions:**

Due to its measurement speed, TLS provides a method to effortlessly capture large amounts of detailed structural forest information, and consequent proxy data for tree and forest processes, at a far wider spatial scale than is feasible with manual measurements. Issues with measurement precision and occlusion of laser beams before they reach their target structures continue to reduce the accuracy of TLS data, but the limitations are counterweighted by the measurement speed that enables large sample sizes. The currently high time-cost of analysing TLS data, in turn, is likely to decrease through progress in automated processing methods. The developments point towards TLS becoming a new and widely accessible standard tool in forest measurement and modelling.

## INTRODUCTION

Forests have been measured with various types of instruments for centuries. Historically the main motivation has been the estimation of yield in upcoming harvests. With time, the measurement instruments and motivations have evolved. Forest mensuration is no longer just about maximizing profits, but also about detecting and maintaining the well-being of the forest – and by proxy the well-being of nature and the planet (McDowell *et al.*, 2020).

In retrospect, it is easy to pinpoint the massive leaps that forest mensuration has taken in the recent decades. Diameter at breast height (DBH), stem girth, tree height, crown height and spread form the standard set of measurements that have been collected from a single tree with basic instruments for a long time. In addition, an expert can determine the tree species and health status by visual inspection, together with rough stem position within the forest plot. Collecting this set of about ten parameters takes ideally from 5 to 20 min per tree. Collecting information regarding the branching structure of an individual tree in a non-destructive manner has also been possible but would take weeks even with a digitizer and a caliper (Sinoquet *et al.*, 1997). Full structural branch-level scanning of a 1-ha forest plot, in turn, has been estimated to roughly take a team of three people between 3 and 8 d using a single scanner (Wilkes *et al.*, 2017). Thus, it is safe to say that applying terrestrial laser scanning (TLS) to forests was certainly a game changer. The surface of a tree can be sampled over a million times within minutes to capture its full 3D structure (Liang *et al.*, 2016), even at sub-centimetre-level spatial resolution (Campos *et al.*, 2020). Furthermore, the scans contain information about the forest bed and surrounding trees and the understorey as well (Calders *et al.*, 2020).

Another leap has happened more slowly, in small increments in the field of data processing, enabling a high level of automation for TLS point cloud computations. One technique or approach that has gained considerable popularity and seen development in the last decade is quantitative structure modelling (QSM), which was first introduced by Raumonen *et al.* (2013) to describe the result of a tree structure reconstruction procedure from TLS data. Similar algorithms have since been published (Hackenberg *et al.*, 2015; Du *et al.*, 2019; Fan *et al.*, 2020). What they have in common and what sets them apart from previous approaches is the comprehensive nature of the resulting models. Rather than measuring a specific tree property, the aim is to produce a complete model of the tree surface, volume and the topological branching structure, which in turn can be used to derive a vast quantity of physiological proxies and geometric properties.

**Fig. 1.**
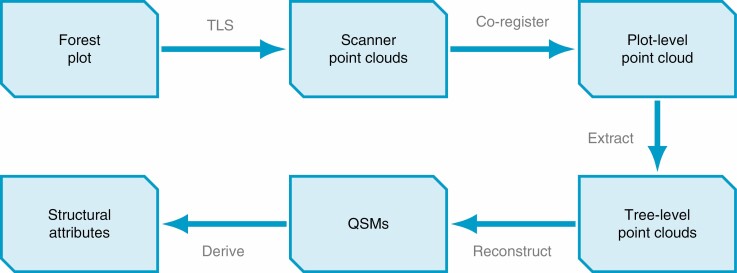
The process of deriving structural tree attributes from a forest plot using terrestrial laser scanning (TLS). Individual scans are co-registered to create a single forest-plot-level point cloud, which is in turn partitioned through tree extraction. The resulting tree-level point clouds are reconstructed as quantitative structure models (QSMs) and can be used to compute countless structural attributes, such as diameters, branching topology and angles, or partial and total volumes.

Figure 1 summarizes the typical workflow of TLS measurements combined with QSM reconstruction, which starts from the scanning of a forest plot and pass through the identification of tree-level point clouds, and construction of individual trees, to the derivation of structural attributes for individual trees (see also fig. 6 by O’Sullivan *et al.*, 2021 in this issue). In essence, multiple scanning positions are used to create point clouds that sample the surfaces of all objects in the study area. This massive point cloud then has to be segmented into individual trees before QSM reconstruction can happen. The resulting comprehensive structure models can be used to derive numerous structural attributes for the trees as individuals, but also for the study area as a whole.

Predicting the future of the TLS research field is hard but perhaps not impossible, given recent trends. The combination of high-detail laser scanning and comprehensive tree reconstruction has obvious benefits compared to manual measurements in terms of measurement speed and structural detail of the collected data. The characteristics of the measuring instruments might change as mobile (MLS) and unmanned, airborne laser scanning (UAV-LS) technologies are explored (Beland *et al.*, 2019), but what seems certain is that going back from TLS to manual measurements alone would not make any sense when considering tree structure. Openly available stand structure scanned by TLS may well become a similar but far more informative standard of forest characterization as are the conventional reports of manually measured tree density, height and stem diameter. The benefit for both forest inventories and forest research is that there will no longer be a limited set of individual measurements of individual sample trees, but rather a comprehensive 3D model – or a time series of 3D models – storing the development of all trees within an entire forest stand and providing data to access countless tree and stand properties applicable to many research questions (Campos *et al.*, 2020).

Here, we will briefly review and discuss recent developments that point towards laser scanning, and the combination of TLS and QSM in particular, becoming a new versatile standard in measuring and modelling multiple features of forest ecosystems. First, we will give a brief overlook of the potential benefits of laser scanning and QSM in the fields of forest mensuration and research of forest structural and functional processes. We then give a brief summary of how the growth in spatial measurements has driven the increase in the possible detail of tree models and allowed forest reconstruction. Predictions regarding the future standard of forest monitoring are thenpresented. Optical sensing technologies and forest reconstruction approaches have certain limitations and some challenges still need to be addressed before comprehensive reconstruction can become a standard, and these are discussed. The role of data analytics in solving some of these challenges is then discussed, followed by conclusions.

Other articles in this issue elucidate multiple sides of current progress in the use and development of TLS and related methods for 3D forest dynamics. They cover TLS sampling strategies (Boucher *et al.*, 2021), automatic tree segmentation from MLS data (Bienert *et al.*, 2021), branch (Hu *et al.*, 2021) and above-ground (Demol *et al.*, 2021; Kükenbrink *et al.*, 2021) biomass estimation, TLS data processing (Martin-Ducup *et al.*, 2021), effects of species mixing on stand productivity (Pretzsch and Schütze, 2021), tree crown interactions (van der Zee *et al.*, 2021), and using TLS for developing models to investigate ecological and evolutionary processes (O’Sullivan *et al.*, 2021).

## POTENTIAL BENEFITS OF LASER SCANNING IN FOREST PROCESS RESEARCH

Alternative laser scanning technologies enable characterization of the 3D details of forest structure at multiple scales (Beland *et al.*, 2019). Airborne laser scanning (ALS) from aircraft quickly collects an estimate of the structure and amount of forest vegetation at the scale of thousands of square kilometres (Wulder *et al.*, 2012), and can be complemented by a smaller, forest-plot-level scale with TLS that has the capacity to capture branch-level details of forest structure also under the canopy surface (Calders *et al.*, 2020). The use of UAV-LS provides an additional intermediate scale (Brede *et al.*, 2019). In cities and within urban forests, laser scanning provides a convenient tool for measuring tree attributes with less demand for destructive harvesting (Kükenbrink *et al.*, 2021).

Estimation of forest resources from an economic perspective is one motivation for employing scanning technology (Wulder *et al.*, 2012), but nowadays an increasing number of studies are developing the methods of scanning tree and forest structure as an aid to investigate the processes of forest dynamics and ecosystem function (Orwig *et al.*, 2018; Beland *et al.*, 2019). The structural data themselves or in combination with additional remotely sensed data collected at the time of scanning can be used to provide information on, for example, tree health (Chi *et al.*, 2020), drought stress (Jacobs *et al.*, 2021), diseases (Husin *et al.*, 2020) and leaf water content (Junttila *et al.*, 2018; Elsherif *et al.*, 2019). Combining multiple types of measurements with structural data provides capacity to enhance the models and to analyse forest ecosystem function at far wider scales and with better spatial precision than is feasible with labour-intensive manual measurements (D’Urban Jackson *et al.*, 2020).

The 3D structure of a forest itself serves as a proxy for many variables and processes, because it consists of features such as plant species composition and distribution, spatial arrangement of plant structures, and the shape of the terrain. For example, the species diversity and structural diversity of forest vegetation has obvious links with processes maintaining the biodiversity of other organisms as they predict the availability of food and microclimatic conditions suitable for different organisms (O’Sullivan *et al.*, 2021). Sample studies have linked forest structure with diverse features such as carbon fluxes and storage (Hardiman *et al.*, 2013), spatial patterns of nutrient cycling (de Godoy Fernandes *et al.*, 2021), variation of photosynthetically active radiation (Ma *et al.*, 2021), forest productivity (Pretzsch and Schütze, 2021), composition of bird communities (Hollie *et al.*, 2020), foraging paths of mammals (Moorcroft, 2012), rainfall interception (Yu *et al.*, 2020), risks of fire and wind (Gopalakrishnan *et al.*, 2020; Gale *et al.*, 2021), canopy interactions (van der Zee *et al.*, 2021), and insect abundance (Knuff *et al.*, 2020).

Importantly, laser scanning and associated measurements serve to refine the estimates of parameters and processes in models of 3D forest dynamics that are increasingly being developed for creating scenarios of long-term forest development under changing environmental conditions and forest disturbances (Huber *et al.*, 2020; Ruiz-Benito *et al.*, 2020). Potential benefits of employing the spatial details of stand structure have been demonstrated, for instance, in models of carbon and water fluxes (Thomas *et al.*, 2008; Simioni *et al.*, 2016), species dynamics (Mertes *et al.*, 2020; Petter *et al.*, 2021), growth and yield modelling (Tompalski *et al.*, 2018; Pretzsch and Schütze, 2021), and landscape-scale stand dynamics (Seidl *et al.*, 2012).

Besides other benefits, the use of laser scanning technology has potential to advance the repeatability of experiments and control the influence of subjective decisions during data collection and processing. For example, it costs much less to re-sample a forest area with a new point cloud through a scanner than it would to send an expedition of field workers to manually measure crown details of large trees within a stand. Provision of open access point cloud data can enable other researchers to check the steps of data processing, whihc are still dependent on subjective decisions (Martin-Ducup *et al.*, 2021). Moreover, capabilities for collecting more accurate time series are also improving (Srinivasan *et al.*, 2014), because scanning of structural and spectral data diminishes the need for destructive sampling that simultaneously alters the system status.

## EVOLUTION OF TREE MODELS

One way to fully appreciate the magnitude of the change that laser scanning represents in the measurement of forests is to understand how measurement-based modelling of tree structure has evolved over time (Liang *et al.*, 2016). In the era of manual measurements, combining a DBH measurement with an estimate of tree height, one could approximate a tree as a single cylinder. To consider stem tapering, consecutive diameter measurements were needed along the stem. To minimize the measurements required, allometric models were developed to estimate stem taper based on DBH and tree height measurements.

Stem models are relatively easy to produce even by simple manual measurements, and a tapering stem model might be sufficient for basic yield estimation, but they provide only limited information on the current condition and growth processes of trees within a stand. More detailed measurements of canopy and branch structure have shown that tree species-dependent phenotypic changes associated with crown morphology and branch plasticity can be central in the development of a stand (Kunz *et al.*, 2019; Hildebrand *et al.*, 2021), and probably contribute to the variation of growth responses in different species mixtures (Pretzsch and Schütze, 2021).

### Canopy and branches

Using basic canopy height and width estimates, canopy volume can be estimated, for example as a circular cylinder, cone or an ellipsoid. This level-of-detail geometric model is still used for many applications, and it can be augmented by introducing a heterogeneous density to simulate light penetration within the canopy (Seidl *et al.*, 2012). In reality, the branches are sparse inside any simple geometric canopy volume, leaving most of the volume unoccupied. Furthermore, canopy models based on geometric shapes do not account for individual branches and varying branch size. In more detailed models, a set of cylinders or other basic meshes inside the canopy volume have been applied to simulate individual branches (Kennedy, 2010). Ideally, however, one would want the generated branching structure to match reality, but it is extremely labour-intensive to manually collect the required measurements regarding size, position, orientation and topological relationships of individual branches (Sinoquet *et al.*, 1997).

TLS or UAV-LS point cloud data can be used to derive canopy size and density estimates, but have also enabled comprehensive structure reconstruction – given high-resolution, non-occluded data – where the geometry of individual branches and the topological branching structure are estimated and stored in a detailed model. Stem and branches can be presented as continuous, parametrized surfaces (Yan *et al.*, 2009) or as sets of geometric primitives (Åkerblom *et al.*, 2015). These models do not consider fine structure, such as bark texture, and in particular geometric primitives cannot be used to capture details such as bulges or crevices. However, comprehensive fine structure reconstruction is beyond the capabilities of any technology at the moment – especially for standing trees.

### Foliage

Individual broadleaves or needles form the foliage cover of a tree. In a healthy tree, most branches contain foliage and trees can have hundreds of thousands of broadleaves or millions of needleleaves, thus making measuring leaf cover parameters extremely challenging (Wu *et al.*, 2020). The notion is complicated further when considering the dynamic nature of the geometry of a broadleaf that is increasing in size during a growth season. Over shorter time frames, wind, rain and even cloudiness can affect the leaf geometry during a day, depending on the time of the day, or even within seconds (Vogel, 2009). An ideal leaf cover model would contain the position, orientation, shape and size of each individual leaf as a function of time, together with information regarding where leaves connect to the woody structure. It is safe to say that we are still far from having a reconstructive, measurement-based approach that would produce such a comprehensive model.

TLS has provided a new way to estimate leaf parameter distributions. The technology has been used to estimate properties, such as density (Grau *et al.*, 2017), orientation (Zheng and Moskal, 2012) and leaf size (Hétroy-Wheeler *et al.*, 2016). Given estimates of the leaf parameter distributions, they can be converted to individual leaves with a fixed geometry by sampling. An algorithm and an open-source implementation for this purpose – together with intersecting geometry prevention – were presented by Åkerblom *et al.* (2018). If QSM from TLS data and leaf parameter distribution estimation and leaf cover generation could be combined so that only a single leaf-on point cloud were required, one could essentially reconstruct a complete snapshot of all the above-ground parts of a tree. Currently, this is not possible as leaves occlude the woody parts, preventing accurate woody structure reconstruction (see ‘Occlusion’ below).

## STANDARD FOR THE FUTURE?

With all the benefits, such as increased capture speed and measurement resolution together with the ability to reconstruct comprehensive models, it is very likely that in particulare the combination of TLS (including UAV-LS and MLS) and QSM will become a widely adopted new standard in forest measuring and modelling, because it allows measurements at the resolution of centimetres at the reasonably wide hectare scale (Beland *et al.*, 2019). Certainly, there is no going back to digitizers due to their slow collection time, and as other structural properties of trees can be estimated accurately from point cloud data or QSM, performing other manual measurements of structure one tree at a time also seems unlikely. There are downsides to optical approaches, but even with them TLS seems more efficient than any current alternative due to the amount of data captured in a short time.

### Automation and real-time processing

However, one current bottleneck in the use of TLS is the technically demanding and time-consuming process of reconstructing QSMs and other meaningful structural information from the captured point clouds. At present, there are numerous published approaches to reconstructing QSMs – TreeQSM (Raumonen *et al.*, 2013), SimpleTree (Hackenberg *et al.*, 2015) and AdTree (Du *et al.*, 2019) to name a few – and probably more on the way. All the listed methods only operate with a tree-level point cloud. These solutions differ with regard to algorithm level, input parameters, documentation level, dependencies and the platform they run on. In particular, the differences in the input parameters can make it hard for a novice user to select a method and utilize it properly.

To improve the user experience and approachability of QSM reconstruction to users from all fields, a black-box solution, which performs automated tree extraction from forest-plot-level point clouds in a standardized manner, would clearly be ideal, as manual extraction can still be extremely time consuming. For example, Martin-Ducup *et al.* (2021) report different steps of reconstruction as taking close to 2 months for a 1-ha tropical forest plot. However, it seems that one-step solutions are not far in the future as semi-automated approaches are already providing meaningful results (Martin-Ducup *et al.*, 2021). When one-step solutions become operational, they will enable a move towards automatic, comprehensive, massive-scale forest reconstruction, as already outlined using another optical measurement technology (Fujimoto *et al.*, 2019).

When a fully automatic solution is available, it can be augmented further with tools such as species and tree health detection. The former has already been shown to be possible with machine learning, given suitable training data (Åkerblom *et al.*, 2017; Terryn *et al.*, 2020), and the prerequisites for the latter have also been demonstrated (Husin *et al*., 2020; Jacobs *et al.*, 2021). In theory, the end-user could input point cloud data and be presented with a report containing stem counts per species, together with an estimate of possible damage or threats for that forest plot. Ideally, even management suggestions could be computed based on the reconstructed models and simulations.

**Fig. 2.**
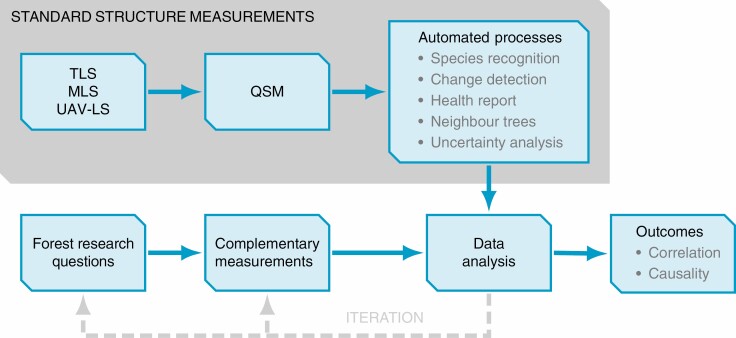
Sketch of a new standard for forest research planning and execution as a flowchart. The standardized structure measurements (grey background) remain constant regardless of the original or revised research questions. The comprehensive structure models and properties derived from them through the automated processes can be exactly the same for multiple different types of studies. The structure data can be combined with optional complementary measurements to perform correlation or causality analysis between any of the measured or derived attributes or, for example, a theoretical model.

In practice, for forest-related research, the standardization of reconstruction can mean the separation and independence of structural measurements with respect the research questions (Fig. 2). Regardless of the focus and aim of the study, structural measurements in the form of QSMs, reconstructed from some terrestrial data source, could be provided as open data in a standard format, which the users can use to extract the desired measures depending on the research question. This means that due to the comprehensive nature of QSMs, the structural measurement component will remain exactly the same and require the same effort regardless of whether the subject of the study is, for example, DBH alone or full canopy structure. Furthermore, collections of QSMs can be automatically processed to derive further information such as tree species and health information, to detect structural changes (see ‘From static to dynamic’ below) and to analyse shadowing and tree competition with neighbouring QSMs.

Complementary measurements pertaining to the research questions, such as soil or bark samples, are still collected in the same way and combined with the structural measurements for data analysis and hypothesis testing, using for instance markers discernible in TLS data or high-precision GPS to determine their positional information. Should iteration be required, the research questions can be modified or additional measurements carried out, while relying on the same standard structure measurements. With open QSM data storing and sharing, it is also possible to conduct studies without the need to perform scanning and reconstruction, but rather use the standard format data from others. Over time and given the current research trends, the library of reconstructed QSMs would have potential to expand rapidly, enabling many types of high-detail simulations of forest dynamics (Calders *et al.*, 2018; Liu *et al.*, 2019; O’Sullivan *et al*., 2021).

### Scanning to reconstruct

It should be remembered that QSM reconstruction can only be as good as the input point cloud data. Thus, when collecting TLS measurements, the upcoming data processing should be considered in planning and *in situ* (Boucher *et al.*, 2021). The data requirements are vastly different when TLS is utilized for stem counting, DBH or gap fraction estimation, compared with input data for comprehensive stand reconstruction (Beland *et al.*, 2019). Scanning practices should be developed especially for QSM reconstruction, and scanner operators should always be aware of what the collected data will be used for. Minimum requirements of resolution could be defined for standard format data.

With increasing data processing capabilities, it will soon become possible to analyse point cloud data in the field while collecting. By identifying individual trees and analysing local coverage and occlusion within their canopies from completed scans, it will be possible to determine where the scanner should be moved next. This type of a concept has already been studied by Li *et al.* (2020) through simulations. Available computing power is already sufficient with modern laptops, so it is only a matter of implementing a combination of automatic co-registration and occlusion analysis. Real-time processing would also mean that redundant data could be detected and prevented or removed while measuring (Boucher *et al.*, 2021).

### From static to dynamic

The section ‘Evolution of tree models’ detailed how increasingly accurate reconstructions of tree geometry and topology have become readily available. However, tree structure without a larger context is quite limited. How that structure came to be, what is the current status of the tree and how the tree will evolve with time are all questions that cannot be answered accurately based on a single structural snapshot of an individual tree. Therefore, to create context, the next logical step is to reconstruct several of these snapshots over a longer period of time and combine them with concurrent structural snapshots of the entire stand surrounding the tree, in order to detect dynamic and interactive changes in the development of the whole tree community (Campos *et al.*, 2020; Yrttimaa *et al.*, 2020*a*).

All the technology is already available as it is the same as for static structure. Advances are only required regarding the sampling procedures and the consequent data processing side for identifying the topologically and geometrically matching parts of two reconstructed structure models (Guan *et al.*, 2020). The analysis for detecting changes in branch radii, lengths and angles should then be trivial. In fact, the concept of QSM change detection has already been tested (Kaasalainen *et al.*, 2014).

On the user experience side, growth and decay detection could be included in the proposed one-step software solution. A collection of previously reconstructed QSMs could be given as an optional input, and any newly reconstructed model could be automatically matched to the existing set, based on location or geometric properties, and change detection would be then carried out. The user would receive the new QSM of each tree and a report on how the structure has evolved since the last reconstruction. It should be noted that the scanning conditions (see ‘Scanning conditions’ below) of the repeated scans should be as similar as possible, or otherwise their difference would have to be accounted for while detecting changes.

The move from studying static tree structures into structural dynamics would enable numerous correlation and causality studies. For example, given measurements of climate or soil conditions at multiple times, and corresponding tree reconstructions, the connection between the change in those measurement values and the changes in tree structure could easily be studied. Similarly, the effects of various forest management strategies would be accurately quantifiable (Camarretta *et al.*, 2020).

## REMAINING CHALLENGES AND LIMITATIONS

### Occlusion

Occlusion is a fundamental limitation of any optical sensing technology. Objects and surfaces can only be detected if they are seen. On the other hand, forests and trees are full of occlusion: trees occlude one another, branches occlude one another and foliage occludes everything. Stem and branch occlusion can be lessened by adding scanning positions with different angles around the tree – or possibly with different heights (Abegg *et al.*, 2017). Depending on the forest plot stem density and other conditions, adding scan positions that decrease occlusion effects may be possible (Yrttimaa *et al.*, 2020*b*; Boucher *et al*, 2021). Furthermore, even with sparse stem densities, it can be difficult to determine *in situ* where to perform the additional scan to minimize self-occlusion within the tree canopy.

Foliage occlusion is much more difficult to account for. With non-evergreen trees, scans can sometimes be performed in the leaf-off season, eliminating foliage occlusion. For other scenarios, data analysis might offer solutions (Hyyppä *et al.*, 2020), as will be discussed in the sections below.

### Scanning conditions

Laser scanning is not well suited to measure moving targets. Thus, scanning of trees in windy conditions is not advised (Vaaja *et al.*, 2016), as branch movement introduces uncertainty in the resulting point cloud. The same applies for rain and fog (Filgueira *et al.*, 2017). For smaller studies it might be possible to wait for suitable weather conditions, but for larger inventories it is not an option. The limitation is fundamental to any laser scanning technology and thus improvements can only be made on the data analysis side.

Jackson *et al.* (2019) showed that reconstructed QSMs can be used to study tree movement and structure deformations under wind flow. Starting with windy-condition point cloud data and a crude reconstruction, it would be possible to simulate the movement of the tree under the recorded wind conditions. An uncertainty value could then be determined for individual range measurements, and uncertain points would be ignored or corrected, providing an improved dataset for final QSM reconstruction.

### Shared format

There currently exists no standard format for storing and sharing reconstructed tree and stand structures. This shortcoming slows data processing development and sharing of results. In theory the fix is simple: a standard should be agreed upon and published. However, with so many approaches to reconstructing QSMs, they are not structurally similar. Some contain multiple geometric shapes per branch (Raumonen *et al.*, 2013), while others use only a single parametric surface (Yan *et al.*, 2009). Other challenges include the need for convenience through redundancy and the inclusion of non-structural, application-specific information.

In short, any standard should include only the minimum amount of structural data. Any redundancy should be eliminated to minimize required storage, as convenience data, such as tree-level statistics, can be derived from branch-level data when needed. Information regarding the utilized reconstruction process and expected model applications should be excluded from the structural description, as such information is hard or impossible to standardize.

Solving the issue of structural dissimilarities might also be possible, as recent approaches seem to model the stem and branches as having circular cross-sections. For example, the resulting models from Yan *et al.* (2009) and Du *et al.* (2019) seem different, as the former uses branch-level, parametric surfaces and the latter circular cylinders as geometric primitives, but the underlying data are still the same. Each branch – and stem – is essentially a set of diameter measurements along the length of the branch. Thus, rather than storing cylinder or surface parameters, the diameter measurements could be stored instead. The benefit of this is that in this way the data would be independent of the methodology it was created with, but both types of models could still easily be derived from it. Thus, only branch-level topology data would have to be stored.

On the stand level, an additional standard is required for efficient sharing of collections of QSMs. At a minimum, such a container would hold the absolute position of a reference point on the forest stand, and the position of all individual trees relative to that reference point. The container could then be used, for example, to find neighbouring trees or to build a virtual representation of the entire stand. To minimize redundancy, any stand-level attributes, derivable from the tree models, should be excluded from the standard.

### Taking data analysis to a new level

Even with all the developments in forest point cloud data processing, there remains a lot underutilized potential. Only rough estimates have been given to uncertainty pertaining to tree attributes, such as above-ground biomass or DBH, but what causes the uncertainty? Additionally, if occlusion cannot be overcome, can it at least be quantified? Is simultaneous woody structure reconstruction and foliage cover distribution parameter retrieval possible – even in theory? These are just some of the questions that can be answered by developing new data analysis tools.

### Uncertainty

Given a QSM of a tree, there will be many sources of error and uncertainty for both the geometry as well as the topology (Demol *et al.*, 2021; Martin-Ducup *et al.*, 2021). Starting with point cloud data collection, the TLS instrument will have uncertainty related to range and angle measurements, together with beam divergence-related uncertainty. After the measurement phase, point cloud co-registration has a certain uncertainty, which can actually lead to massive errors in reconstructed branch volumes. Occlusion is another source of uncertainty. Additionally is the uncertainty of the selected reconstruction procedure and the chosen model format, and finally the uncertainty of computing a feature from the resulting model.

With detailed error analysis, it will be possible to study, separate and quantify the level of error relating to each of the phases of tree reconstruction (Demol *et al*., 2021; Martin-Ducup *et al.*, 2021). Extensive simulations and scans in laboratory conditions could be used to give theoretical limits for each source of uncertainty, independent from one another, forming a sort of uncertainty distribution for the error sources. If successfully completed, each reconstructed tree model and attributes derived from them would have an attached total uncertainty and error limits.

### Analysing occlusion

By design, laser scanning and tree reconstruction focus on parts of the tree that are seen. This idea is fundamental and even built-in to the term ‘point cloud’, meaning a collection of discrete and localized laser beam intersections. In a wider context, laser scanning produces a set of rays that traverse the volume containing a single tree. As a simplification, for each ray at an interception point the probability of an existing surface is one and before it, the probability is zero. After an interception the view is occluded – at least partially – and the probability is between zero and one. Thus, the set of traversed rays can be converted into a 3D probability distribution for occlusion analysis.

Given a probability distribution of volume, an upper limit for occluded volume can be computed. Furthermore, the estimate can be improved by considering what kind of branching architecture and surface geometry is realistic or probable at any given location (Lecigne *et al.*, 2021). Example constraints could include an upper limit for the branch radius as a function of distance from the stem, and the requirement of an occluded path, connecting the missed branch to the detected, reconstructed surface.

### Inverse approaches

As mentioned in the ‘Foliage’ section above, multiple methods have been developed to measure various foliage distribution parameters with TLS. Such an approach is a called a forward approach, as the quantity being studied is measured directly. In an inverse approach the measurements are used to indirectly infer what type of an unknown object or – in this application, a distribution – resulted in those measurements. For example, given a known woody surface and TLS measurement setup, it would be possible to generate foliage covers with varying distributions and then simulate TLS for those candidate leaf covers. The resulting simulated point clouds could then be compared to the real point cloud data, allowing the determination of the real foliage distribution parameters, by selecting the optimal candidate by some suitable metric.

In essence, the proposed inverse approach would use machine learning to determine properties not directly accessible from the point cloud data (Xi *et al.*, 2020). Training data could be collected through simulations or in controlled stand conditions, and they would contain a mapping between measured quantities, such as local point cloud variations and the unknown, underlying quantities such as leaf size and orientation distribution parameters. The accuracy of the methodology can be uncreased by introducing a priori information, regarding for example expected leaf sizes.

## CONCLUSION

In the last decades, the application of laser scanning instruments for forest mensuration have grown significantly in popularity, enabling numerous new approaches for forest research as described here. Even faster – in less than a decade since its introduction – the concept of QSM has morphed from a borderline approach into one of the most widely utilized data processing methods for point clouds. More broadly, this has represented the switch from individual tree structure measurements to comprehensive tree reconstruction.

Although TLS- and QSM-based approaches have taken a foothold in forest structure and process research, they have not yet become *the* standard. However, their standardization seems not to be far into the future as the TLS instruments become more common, cheaper and easily portable (Wang *et al.*, 2019). Novel approaches and improvements to existing solutions for comprehensive tree reconstruction seem to be constant (Bienert *et al.*, 2021), and it is only a matter of time when a fully automatic, parameter-free method will be presented, operating on forest-plot-level point cloud data. Such a black-box solution will certainly have its appeal, but it is also important to understand the built-in assumptions to fully comprehend the behaviour and limitations of any algorithm. A practical way to accomplish this would be to start following the development of existing methods, giving feedback and bridging the gap between algorithm developers and forest researchers in the process, thus ensuring the suitability and usability of the end result.

Separate from reconstruction development, it is also the perfect time to start planning what will happen when comprehensive reconstruction becomes standard. How will research change when it can be assumed that detailed QSMs will be available for any forest site scannable by TLS, MLS (Bienert *et al.*, 2021) or UAV-LS (Brede *et al.*, 2019) technologies? Will we still use traditional properties, such as DBH, to describe structure, or will they be replaced by other quantities that are more representable and not just accessible? Tree competition-related canopy properties (van der Zee *et al.*, 2021) and tree species (Terryn *et al.*, 2020) can already be automatically derived from TLS data or QSMs, but what other characteristics can be developed for the point cloud data processing chain?

When looking at a QSM of a single tree, it is often easy to see that there are errors, especially when there are issues with the input TLS data quality. What should be remembered though is that, even with those errors QSM reconstruction is probably the most accurate description of the entire structure of that tree. It might not be sufficient for every application, but it is certainly more than a traditional combination of a DBH value and a possible estimate of tree height. Thus, rather than focusing on the current shortcomings of TLS-based comprehensive reconstruction, a more productive approach would be to try to overcome them. This can mean either focusing on improving reconstruction accuracy (Fan *et al.*, 2020), or developing complementary techniques utilizing the same input data (Hu *et al.*, 2021) or additional field measurements (Yrttimaa *et al.*, 2020*b*). Any additional structural measurements can in theory be used as guiding constraints for the reconstruction. An interesting study would be on what type of extra measurements would improve reconstruction accuracy the most.

In the end, for a new standard to be created it must be accepted that new technology is changing forest measurements for good. This does not mean that the results will ever be perfect – just continuously improving.

## FUNDING

M.Å. was funded by Academy of Finland’s project Centre of Excellence in Inverse Modelling and Imaging.
